# Development and Application of Specific Cytokine Assays in Tissue Samples from a Bottlenose Dolphin with Hyperinsulinemia

**DOI:** 10.3389/fendo.2013.00134

**Published:** 2013-10-01

**Authors:** Kirsten C. Eberle, Theresa E. Waters, Eric D. Jensen, Stephanie K. Venn-Watson, Randy E. Sacco

**Affiliations:** ^1^Ruminant Diseases and Immunology Research Unit, National Animal Disease Center, Agricultural Research Service, United States Department of Agriculture, Ames, IA, USA; ^2^Molecular Cellular and Developmental Biology Graduate Program, Iowa State University, Ames, IA, USA; ^3^Immunobiology Graduate Program, Iowa State University, Ames, IA, USA; ^4^Navy Marine Mammal Program, San Diego, CA, USA; ^5^Translational Medicine and Research Program, National Marine Mammal Foundation, San Diego, CA, USA

**Keywords:** *Tursiops truncatus*, hyperinsulinemia, diabetes, inflammation, cytokines, real-time PCR, immunohistochemistry

## Abstract

Chronic inflammation has been associated with insulin resistance and type 2 diabetes (T2D) in humans. Postmortem hepatic and splenic tissue from a 46-year-old geriatric male bottlenose dolphin (*Tursiops truncatus*) with insulin resistance (chronic hyperinsulinemia with hyperglycemia), chronic inflammation (white blood cell count greater than 12,000 cells/μL), and mild fatty liver disease was evaluated for elevated pro-inflammatory mediators. Cytokine mRNA expression in postmortem hepatic and splenic tissue, as determined by real-time PCR, included an array of cytokines: TGF-β, TNF-α, IFN-γ, IL-2, IL-4, IL-10, IL-12p40, IL-13, and IL-18. Values from this dolphin were compared to a younger reference dolphin with no known chronic metabolic perturbations or inflammation. Levels of TGF-β, TNF-α, and IL-4 were higher in the case dolphin’s liver compared to that of the reference dolphin. In the case dolphin’s spleen, IL-10 and IFN-γ mRNA was upregulated while IL-4 was less than the reference dolphin. IL-18 and IL-13 were upregulated in both tissues. Fluorescent immunohistochemistry (IHC) utilized the following antibodies: anti-porcine IL-6, anti-bovine IFN-γ, IL-4, and IL-10, anti-human TGF-β, anti-ovine IL-1β, and anti-dolphin IL-8. Fluorescent IHC in spleen from the case dolphin revealed staining of IL-4, IL-6, IL-8, and TGF-β throughout the tissue. IL-10 and IFN-γ were seen to predominate in areas surrounding the follicles of splenic tissue. This is the first characterization of cytokine levels in dolphin hepatic and splenic tissue. While there are limitations to a case study, this report of inflammatory biomarkers in tissues of a dolphin with insulin resistance and fatty liver disease are similar to those observed in human patients.

## Introduction

Chronic inflammation is associated with, and can be a driver of, insulin resistance and type 2 diabetes (T2D) in humans (1–6). Primary cytokines involved in insulin resistance-associated inflammation are tumor necrosis factor alpha (TNF-α) and interleukin-6 (IL-6) (7–10). Additional cytokines associated with metabolic perturbations include IL-1β, IL-8, IL-10, transforming growth factor-beta (TGF-β), and nerve growth factor (11). Neutralization of TNF-α in obese-diabetic rodents lead to elevated insulin-stimulated peripheral glucose uptake and an overall increase in insulin sensitivity in adipocytes as compared to the controls (12–14). TNF-α signaling induces production of suppressor of cytokine signaling (SOCS), I kappa B kinase beta (Ikkβ), C-jun N-terminal kinase 1 (JNK1), and nitric oxide, which block insulin receptor substrate signal transduction of the insulin receptor (15). While obesity is a commonly researched underlying factor related to chronic inflammation and insulin resistance in humans, inflammation can also be an independent predictor of insulin resistance in non-obese people (16).

Bottlenose dolphins (*Tursiops truncatus*) are emerging as a model for T2D (17). After dolphins consume a high-protein diet, plasma glucose levels rise for up to 5 h, and oral administration of dextrose causes a sustained period (up to 10 h) of hyperglycemia. Further, dolphins are susceptible to diseases and conditions associated with insulin resistance, including chronic inflammation (18). Dolphin-specific cytokine assays were developed to evaluate cytokine activity in this species. The current case study applied real-time polymerase chain reaction (PCR) assays and fluorescent immunohistochemical staining in postmortem hepatic and splenic tissue from a geriatric, 46-year-old dolphin with a history of insulin resistance (chronic hyperinsulinemia with hyperglycemia), chronic inflammation, and fatty liver disease. In humans, hyperinsulinemia and chronic inflammation have been correlated with insulin resistance (1, 19). Specifically, middle-aged males and females with metabolic syndrome have an increased risk for T2D (20). This dolphin died from anaphylactic shock, and related immunologic changes were also expected. Immune values from the case dolphin were compared to those from a reference dolphin with no metabolic perturbations. The presence and quantity of cytokines in the spleen and liver were determined using real-time PCR assays and fluorescent immunohistochemistry (IHC) for mRNA and protein, respectively. This study represents the first evaluation of cytokines in bottlenose dolphin hepatic and splenic tissue.

## Materials and Methods

### Subjects and sample collection

Fresh postmortem tissue from liver and spleen were collected opportunistically from two bottlenose dolphins in the same managed population. Spleen was selected as a representative secondary lymphoid tissue. Samples were snap frozen and stored frozen for later experiments. The case dolphin was a geriatric 46-year-old male with insulin resistance (chronic glucose >122 mg/dL and 2 h postprandial insulin >20 μIU/mL) that died unexpectedly from anaphylactic shock. The reference dolphin was a juvenile male with no evidence of liver disease that died acutely from non-infectious and non-toxic causes. This dolphin had no history of elevated liver enzymes or iron, and no significant findings in hepatic, splenic, or pancreatic tissue upon histologic examination. Historical medical records, including blood data and histologic reports, from the case animal were also reviewed.

### Total RNA extraction and cDNA synthesis

Tissues were removed from RNA *later* Solution (Qiagen) and minced before adding TRIzol Reagent (Invitrogen). Phase separation was accomplished by addition of chloroform and the samples centrifuged at 3400 rpm for 1 h at 4°C. The supernatant was collected and isopropyl alcohol added to precipitate the RNA. After centrifugation at 3400 rpm for 30 min at 4°C, the supernatant was discarded and the RNA pellet was washed with 75% ethanol and centrifuged. The supernatant was discarded and the RNA pellet allowed to dry before being dissolved in RNase-free water. DNase digestion was accomplished using the Ambion TURBO DNA-*free* kit. RNaseOUT (Invitrogen) was added to each sample. First-strand cDNA synthesis was successful with 300 ng of RNA transcribed using Random Primers and dNTP Mix followed by 5× First-Strand Buffer, 0.1 M DTT, and Superscript III Reverse Transcriptase (Invitrogen).

### Real-time PCR assay

Polymerase chain reaction primers were developed specifically for SYBR Green quantification. All sequences were based on previously published information (21). Briefly, SYBR Green Mastermix (Invitrogen) was added to 10 μM of both the forward and reverse primer (Integrated DNA Technologies), dH_2_O, and cDNA template. The amplification conditions were: 95°C for 10 min, followed by 40 cycles of 95°C for 15 s and 60°C for 1 min, and a final dissociation step. Plates were run on an Applied Biosystems 7300 Real-Time PCR System. Relative gene expression was determined using the 2^−ΔΔCt^ method (22). S-9 was used as the reference gene.

### Fluorescent IHC

Snap-frozen sections of dolphin spleen in optimal cutting temperature (OCT) compound (Tissue Tek) were cut, 5 μm thick, with a cryostat and placed on clean poly-l-lysine coated glass slides. The tissue was immediately fixed with an equal ratio of acetone to methanol for 10 min. A liquid-wax boundary was drawn around the tissue with a Super PAP Pen (Invitrogen) to protect against reagent loss. Tissues were blocked with Background Buster (Accurate Chemical and Scientific Corporation) and avidin, if applicable, from an Avidin/Biotin Blocking Kit (Vector) for 45 min. Following a wash with 1× Tris buffer, primary cytokine antibodies were added at varying concentrations and incubated at room temperature for 1 h, unless noted (Table 1). After washing with 1× Tris buffer, appropriate secondary antibodies conjugated to either Alexa Fluor 488 or Alexa Fluor 594 (Invitrogen) were then incubated at a concentration of 5 μg/mL for another hour at room temperature in the dark. Ki67 antibody (Thermo Scientific) was used at a 1:500 dilution and incubated for 1 h with both primary and secondary antibody as described above. In some cases, CD2 was costained as described previously at a concentration of 1:100. The slides were washed a final time with 1× Tris buffer and allowed to dry. Sections were mounted using VECTASHIELD HardSet Mounting Medium with DAPI (Vector) and coverslipped. The mounting medium was left to cure overnight until visualization the following day using a Zeiss Axio Scope.A1 microscope.

**Table 1:** **Primary antibodies used for fluorescent IHC**.

Cytokine specificity	Type of antibody	Species	Dilution	Source
CD2	mAb (IgG1)	Dolphin	1:100	UC Davis UC-F21.C
Ki67	pAb	Multiple species	1:500	Thermo Scientific PA5-19462
IL-1β	pAb	Ovine	1:50 overnight	Serotec AHP 423
IL-4	mAb (IgG2a)	Bovine	1:25	Serotec MCA2371
IL-6	pAb	Porcine	1:50	Thermo Scientific PP690
IL-8	pAb	Dolphin	1:200	Kingfisher Biotech, Inc. PB0377P
IL-10	mAb (IgG2b)	Bovine	1:50 overnight	Serotec MCA2110
IFN-γ: biotin	mAb (IgG1)	Bovine	1:50	Serotec MCA1783B
TGF-β	mAb (IgG1)	Human	1:50 overnight	Serotec MCA797

## Results

### Dolphin clinical results

**Figure 1:**
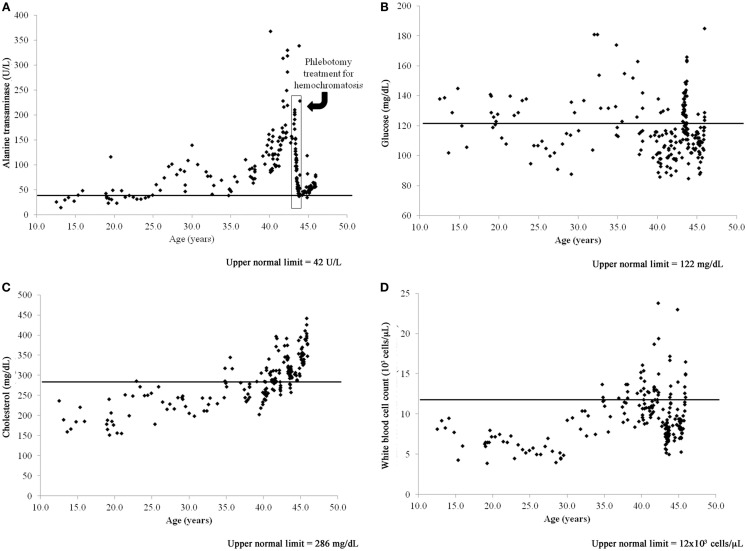
**Time series of blood value changes in the case bottlenose dolphin (*Tursiops truncatus*) with treated hepatic iron overload and chronic, postprandial hyperinsulinemia, and mild fatty liver disease: (A) alanine transaminase, (B) glucose, (C) cholesterol, and (D) white blood cell count**.

The case dolphin had a clinical history of chronic, high liver enzymes (alanine transaminase >42 U/L, aspartate transaminase >263 U/L, and gamma-glutamyl transpeptidase >44 U/L) and serum iron that began at the age of 25 years and peaked at 42 years [Figure 1A; (23)]. A liver biopsy at that time demonstrated that this dolphin had diffuse, moderate hemosiderosis, mild multifocal vacuolar degeneration, mild amounts of granular intracellular iron in hepatocytes, and moderate amounts of intracellular iron visualized via staining in the Kupffer cells. The case dolphin was diagnosed with hemochromatosis (iron overload) and successfully treated with a 20-week course of phlebotomy (23). The dolphin was 43 years old when treatment was completed; all liver enzymes and serum iron values had returned to normal levels for this dolphin population (24).

The case dolphin had repeatedly high 2-h postprandial insulin levels (mean = 31 ± 11 μIU/mL and median = 28, range 17–52 μIU/mL, based upon 16 sampling dates), chronically high glucose (>122 mg/dL), white blood cell counts (>12,000 cells/μL), and high and rising cholesterol (>286 mg/dL) based upon reference values for this dolphin population (25). Both high glucose and cholesterol remained present after treatment for hemochromatosis (Figures 1B,C). When the dolphin died at 46 years old, he had no evidence of hepatic iron deposition, supporting that iron overload in the liver had been successfully treated. Postmortem liver had mild, subacute periportal lymphoplasmacytic inflammation; cholestasis; and mild diffuse, hepatocellular fatty change confirmed to be lipid type with oil red O staining (26). Upon histologic evaluation, there were no significant findings in the pancreas or spleen.

### Cytokine gene expression levels in a dolphin with hyperinsulinemia as compared to a reference

**Figure 2:**
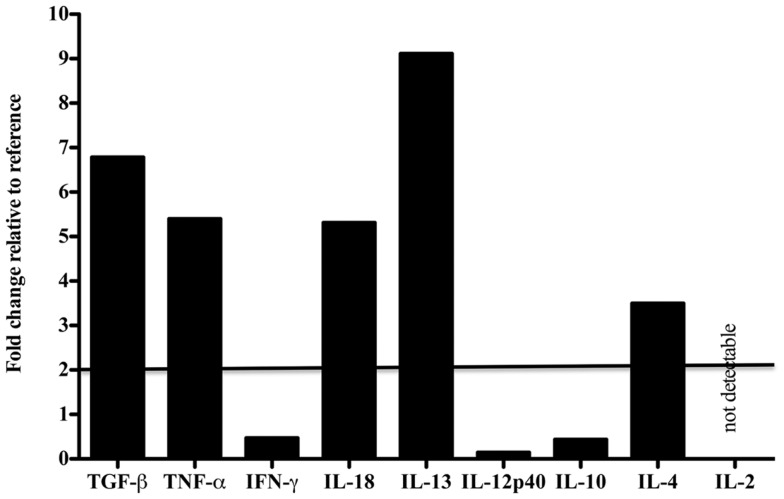
**Cytokine message levels in the liver of a dolphin with hyperinsulinemia**. In the liver, each target gene was determined by real-time PCR and normalized first to S-9, a ribosomal control gene to give a ΔCt. The ΔΔCt was determined for the dolphin with hyperinsulinemia by normalizing to a healthy control. The ΔΔCt were transformed (2^−ΔΔCt^) to show the expression of each cytokine message as a fold change. A fold change of 2 or more was considered to be upregulated in the dolphin with hyperinsulinemia.

**Figure 3:**
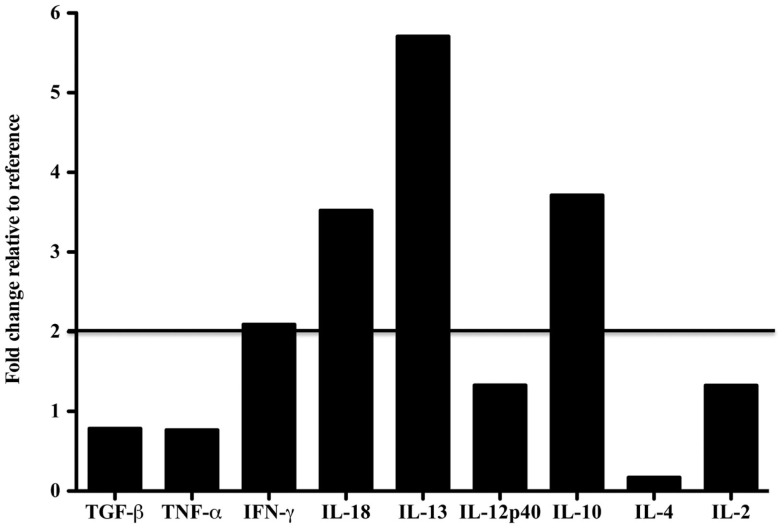
**Cytokine message levels in the spleen of a dolphin with hyperinsulinemia**. In the spleen, each target gene was determined by real-time PCR and normalized first to S-9, a ribosomal control gene to give a ΔCt. The ΔΔCt was determined for the dolphin with hyperinsulinemia by normalizing to a healthy control. The ΔΔCt were transformed (2^∼ΔΔCt^) to show the expression of each cytokine message as a fold change. A fold change of 2 or more was considered to be upregulated in the dolphin with hyperinsulinemia.

Tissue-specific differences in pro- and anti-inflammatory cytokine mRNA expression between the case and reference dolphin were observed by real-time PCR in the liver (Figure 2) and spleen (Figure 3). Levels of TGF-β and TNF-α in the liver were upregulated, but were similar to the reference dolphin in the spleen. IL-18 and IL-13 were upregulated in both tissues. On the other hand, IL-10 mRNA expression was lower than expression in the reference liver while IL-4 was upregulated; the opposite was true in the spleen. IL-12p40 levels were lower in the liver and similar in the spleen compared to the reference dolphin. IL-2 was not increased in the spleen and not detectable in the liver. IFN-γ was lower than the reference dolphin in the liver, but upregulated in the spleen.

### Cytokine expression in the spleen as detected by fluorescent IHC

**Figure 4:**
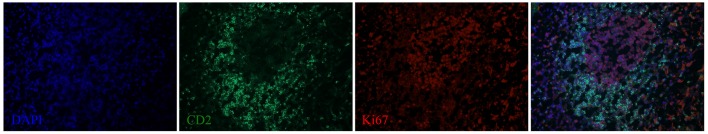
**A germinal center, surrounded by a T-cell zone, in the spleen of a dolphin with hyperinsulinemia**. Dolphin splenic tissue sections were incubated with primary anti-Ki67, followed by anti-dolphin CD2, and nuclear staining using VECTASHIELD mounting medium with DAPI. Original magnification 20×.

**Figure 5:**
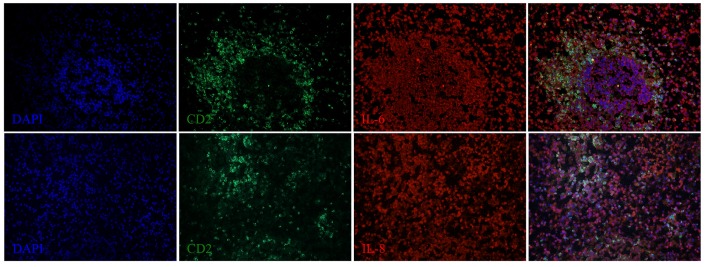
**IL-6 and IL-8 expression in the spleen of a dolphin with hyperinsulinemia**. Dolphin splenic tissue sections were incubated with primary anti-porcine IL-6 or anti-dolphin IL-8, followed by anti-dolphin CD2, and nuclear staining using VECTASHIELD mounting medium with DAPI. Original magnification 20×.

**Figure 6:**
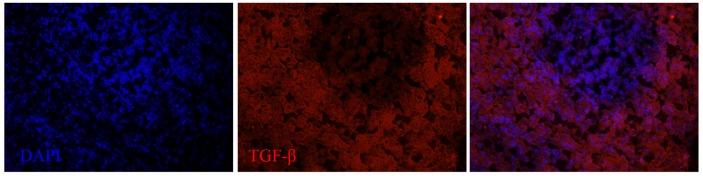
**TGF-β expression in the spleen of a dolphin with hyperinsulinemia**. Dolphin splenic tissue sections were incubated with primary anti-human TGF-β, followed by nuclear staining using VECTASHIELD mounting medium with DAPI. Original magnification 20×.

**Figure 7:**
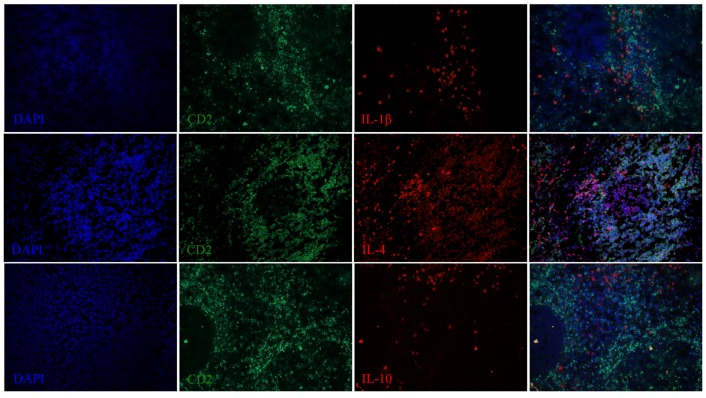
**IL-1β, IL-4, and IL-10 expression in the spleen of a dolphin with hyperinsulinemia**. Dolphin splenic tissue sections were incubated with primary anti-ovine IL-1β, anti-bovine IL-4, or anti-bovine IL-10, followed by anti-dolphin CD2, and nuclear staining using VECTASHIELD mounting medium with DAPI. Original magnification 20×.

**Figure 8:**
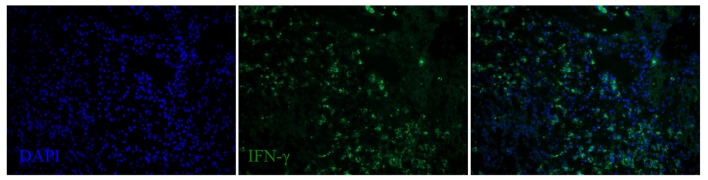
**IFN-γ expression in the spleen of a dolphin with hyperinsulinemia**. Dolphin splenic tissue sections were incubated with primary anti-bovine IFN-γ, followed by nuclear staining using VECTASHIELD mounting medium with DAPI. Original magnification 20×.

We next examined the localization of cytokines in splenic tissue samples from the case dolphin. Fluorescent IHC staining using an antibody to Ki67, which recognizes a nuclear cell proliferation antigen, in the dolphin with hyperinsulinemia showed evidence of multiple germinal centers at various stages of development in the splenic tissue examined, with one representative example shown in Figure 4. Areas of CD2 staining, the T-cell zone, surrounded the germinal centers. Staining of IL-6 and IL-8 was seen in the splenic red pulp, as well as in the germinal centers and T-cell zones (Figure 5), while TGF-β was evidently localized to the red pulp of the spleen (Figure 6). IL-1β and IL-10 staining were seen in the T-cell zone (Figure 7). Two different staining patterns were observed for IL-4; minimal staining was found within the B-cell follicle, while more prominent punctate staining was localized to the red pulp (Figure 7). IFN-γ staining was detected in the T-cell zone and red pulp of the spleen (Figure 8; and data not shown).

## Discussion

Compared to a reference bottlenose dolphin without metabolic perturbations, the current study identified activated cytokine gene expression, including TGF-β, TNF-α, IFN-γ, IL-18, IL-13, IL-10, and IL-4 in the liver or spleen of a geriatric bottlenose dolphin with hyperinsulinemia. The case dolphin has been previously identified as having insulin resistance (chronic postprandial hyperinsulinemia and hyperglycemia) and mild fatty liver disease (17, 23, 26). High white blood cell counts in humans can predict development of T2D, and the clinical profile of the case dolphin, including high white blood cell counts, supports that it had chronic inflammation associated with T2D (27). The source of chronic inflammation was not evident upon histological examination.

This study shows that a dolphin with hyperinsulinemia and metabolic syndrome exhibited similar altered cytokine expression as has been shown in humans and mice with hyperinsulinemia and T2D. Pro-inflammatory cytokines are strong contributors to increased insulin resistance, specifically TNF-α (8, 11, 28, 29). TNF-α interferes with the signaling of the insulin receptor (14). As expected, this dolphin with metabolic syndrome and hyperinsulinemia displayed levels of TNF-α in the liver that were higher than the reference. IL-6 has also been linked to a hyperinsulinemic state (8, 11). Fluorescent IHC in the spleen of the dolphin with hyperinsulinemia did reveal abundant staining for IL-6. In the hyperinsulinemic dolphin, IL-18 mRNA was upregulated in both the spleen and liver. This is consistent with reports in humans that IL-18 predicts the development of T2D (30) and that circulating levels of IL-18 are elevated in patients with T2D as compared to non-diabetic controls (31–33). Due to the pro-inflammatory nature of hyperinsulinemia, it is not surprising that corresponding anti-inflammatory cytokines, IL-4, IL-10, IL-13, and TGF-β, would also be upregulated in an effort to control the inflammation. The case dolphin, however, died unexpectedly from anaphylactic shock, and only one reference dolphin was used. While this is a useful case study, larger scale investigations using more dolphins with and without hyperinsulinemia and metabolic syndrome are needed to further validate this study’s findings. Limitations to such large-scale studies include opportunistic access to properly processed fresh tissues from dolphins of known metabolic status.

Germinal center formation is a characteristic feature of T-cell dependent, B-cell responses and may be an important site of immune dysregulation in certain diseases. Development of splenic germinal centers occurs over a period of days; the follicular dendritic cell network is filled with Ki67^+^ B cells by 7–9 days post-immunization in mice (34). As the cytokine mRNA data suggested the induction of cytokines known to play a role in isotype switching, we examined whether there was evidence of germinal center formation in areas adjacent to the T-cell zone or periarteriolar lymphoid sheath in the spleen. In the present study, the splenic tissue of the case dolphin with hyperinsulinemia was found to contain numerous germinal centers containing Ki67^+^ cells. Interestingly, this is similar to the observation of spontaneous germinal center formation that has been described in strains of mice that develop autoimmune type 1 diabetes (35).

Dolphins are emerging as a model for T2D due to some similarities in metabolic perturbations to humans (17, 24, 26). This study is the first to show that a dolphin with hyperinsulinemia and metabolic syndrome displayed a similar pro-inflammatory state as humans with hyperinsulinemia and T2D. Control tissues from a number of healthy dolphins are needed to make any definitive conclusions based on cytokine protein levels; this would be necessary to determine a baseline expression of each cytokine. It is also pertinent to look specifically at adipocytes and the adipokines they secrete, as the adipose tissue is important in regulating metabolism. Of the cytokines included herein, IL-6 has been shown to be important in hyperinsulinemia in humans and alterations in levels of IL-6 in the dolphin should be examined further. Soluble CD163 (sCD163) was shown to be associated with insulin resistance in humans and may be a more reliable measure of T2D than TNF-α (36); sCD163 secreted by macrophages should be examined further in the dolphin as well. As used in this study, specific anti-ovine, -porcine, -bovine, and -human cytokine antibodies were shown to cross-react with bottlenose dolphin tissues, as similarly described for other cetaceans (37). Reagents for use in dolphins will need to become more available for studying T2D and other diseases. The availability of these reagents and dolphin-specific reagents that we are developing in collaboration with Kingfisher Biotech, Inc., will facilitate studies of immune modulation in managed bottlenose dolphins.

## Conflict of Interest Statement

The authors declare that the research was conducted in the absence of any commercial or financial relationships that could be construed as a potential conflict of interest.
